# CT Enhancement and 3D Texture Analysis of Pancreatic Neuroendocrine Neoplasms

**DOI:** 10.1038/s41598-018-38459-6

**Published:** 2019-02-18

**Authors:** Mirko D’Onofrio, Valentina Ciaravino, Nicolò Cardobi, Riccardo De Robertis, Sara Cingarlini, Luca Landoni, Paola Capelli, Claudio Bassi, Aldo Scarpa

**Affiliations:** 1 Department of Radiology, G.B. Rossi Hospital - University of Verona, Verona, Italy; 2 Department of Radiology, Ospedale Civile Maggiore, Verona, Italy; 3 Department of Oncology, G.B. Rossi Hospital - University of Verona, Verona, Italy; 4 Department of General and Pancreatic Surgery, Pancreas Institute, G.B. Rossi Hospital - University of Verona, Verona, Italy; 5 Department of Pathology, Pancreas Institute, G.B. Rossi Hospital - University of Verona, Verona, Italy

## Abstract

To evaluate pancreatic neuroendocrine neoplasms (panNENs) grade prediction by means of qualitative and quantitative CT evaluation, and 3D CT-texture analysis. Patients with histopathologically-proven panNEN, availability of Ki67% values and pre-treatment CT were included. CT images were retrospectively reviewed, and qualitative and quantitative images analysis were done; for quantitative analysis four enhancement-ratios and three permeability-ratios were created. 3D CT-texture imaging analysis was done (Mean Value; Variance; Skewness; Kurtosis; Entropy). Subsequently, these features were compared among the three grading (G) groups. 304 patients affected by panNENs were considered, and 100 patients were included. At qualitative evaluation, frequency of irregular margins was significantly different between tumor G groups. At quantitative evaluation, for all ratios, comparisons resulted statistical significant different between G1 and G3 groups and between G2 and G3 groups. At 3D CT-texture analysis, Kurtosis resulted statistical significant different among three G groups and Entropy resulted statistical significant different between G1 and G3 and between G2 and G3 groups. Quantitative CT evaluation of panNENs can predict tumor grade, discerning G1 from G3 and G2 from G3 tumors. CT-texture analysis can predict panNENs tumor grade, distinguishing G1 from G3 and G2 from G3, and G1 from G2 tumors.

## Introduction

Tumor grade is the most important prognostic factor in pancreatic neuroendocrine neoplasms (PanNENs)^1–3^. According to the 2010 World Health Organization (WHO) classification^4^, PanNENs are classified into three groups based on their proliferative activity, expressed as mitotic count or Ki67 index: grade 1 (<2 mitoses/2 mm^2^/and/or Ki67 index ≤2%); grade 2 (2–20 mitoses/2 mm^2^ and/or a Ki67 index between 3 and 20%) and grade 3 (≥21 mitoses/2 mm^2^ and Ki67 index >20%). According to European Neuroendocrine Tumor Society (ENETS) recommendations^1^, the assessment of tumor grade is essential for prediction of prognosis and choice of the proper treatment strategy.

The assessment of tumor grade can be achieved in an invasive way with biopsy or after surgery with surgical specimen histopathological analysis. Several studies attempted to identify radiological predictors of malignancy for PanNENs^5–11^, including tumor conspicuity in MDCT images, CT perfusion parameters, and values on MRI, including values in ADC and DWI images. Recently, computed analysis of imaging data has gained increasing interest due to the potential of predicting the aggressiveness of PanNENs^12^. Despite very few literature data, the computed texture analysis of computed tomography (CT) data seems to be able to provide predictive metrics for several pathological features. For example, Lubner MG *et al*.^13^ reported an association between CT texture features with pathological features and clinical outcomes in patients with metastatic colorectal cancer.

The aim of this study was to evaluate PanNENs grade prediction possibility by means of CT qualitative and quantitative analysis as well as of CT 3D texture analysis.

## Materials and Methods

### Patients population and inclusion criteria

All procedures performed in studies involving human participants were in accordance with the ethical standards of the institutional and/or national research committee and with the 1964 Helsinki declaration and its later amendments or comparable ethical standards.

Informed consent was obtained from each individual included in the study. This retrospective study was approved by institutional review board of the University of Verona.

A review of our radiological, surgical and histopathological databases for the period between January 2009 and September 2016 identified all patients with PanNENs. The review board of GB Rossi University Hospital and Ospedale Pederzoli approve to merge and review the data.

Patients were included if fulfilled the following criteria: (a) histopathologically-proven PanNEN; (b) availability of Ki67% values; (c) availability of a pre-treatment CT examination.

Exclusion criteria were: lack of pre-treatment CT examinations and/or absence of Ki67% values.

### Image analysis

CT images were retrospectively reviewed by two radiologists in consensus expert in abdominal radiology, blinded to histopathological features of PanNENs. Pre-contrast, pancreatic phase and portal phase images were retrieved from PACS and transferred to a personal computer for image analysis. Three different image analyses were conducted: qualitative analysis, quantitative analysis, and texture analysis.

### Qualitative imaging analysis

Qualitative image analysis included: (a) margins of the lesion (sharp or irregular); (b) presence of intratumoral hypodense areas; (c) presence of calcifications; (d) dilation of the main pancreatic duct (MPD, >3 mm) and/or common bile duct (CBD, >1 cm); (e) involvement of peri-pancreatic vessels; (f) presence of liver metastases; (g) tumor enhancement compared to pancreatic parenchyma (hyper-, iso-, or hypo-enhancing); (h) presence of inhomogeneous enhancement.

### Quantitative imaging analysis

A ROI was drawn within the tumor on the CT slice in which the lesion showed its larger size (HU_tumor_), both in the arterial and in the portal phases. Care was taken during ROI positioning, in order to avoid adjacent vessels and calcifications. Circular ROIs were also placed in the adjacent pancreatic parenchyma (HU_pancreas_), within the aorta (HU_aorta_) and the portal vein (HU_portal_) for data comparison. ROIs were drawn on the CT image in which the tumor showed its greatest detectability and then automatically copied to the corresponding image on other CT phases. Owing to the variability between examinations and between patients, all values were considered as the ratio between tumor metrics and those of the adjacent parenchyma and the reference vessels.

The following values were considered: (a) relative enhancement ratio, expressed as tumor density compared to the adjacent parenchyma (HU_tumor_/HU_pancreas_) both in pancreatic (*tumor parenchyma ratio 1*) and in portal (*tumor parenchyma ratio 2*) phases; (b) standardized enhancement ratio, expressed both as the tumor density on the pancreatic phase compared with aortic enhancement (HU_tumorART_/HU_aorta_) (*tumor arterial ratio*) and the tumor density on the portal phase compared with portal enhancement (HU_tumorPORT_/HU_portal_) (*tumor venous ratio*); (c) *tumor permeability ratio 1*, defined as [(HU_tumorART_ + HU_tumorPORT_)/HU_aorta_]; (d) *tumor permeability ratio 2*, defined as [(HU_tumorPORT_ − HU_tumorART_)/HU_aorta_]; (e) *tumor permeability ratio 3*, defined as (HU_tumorART_/HU_tumorPORT_)].

### Texture imaging analysis

**Figure 1:**
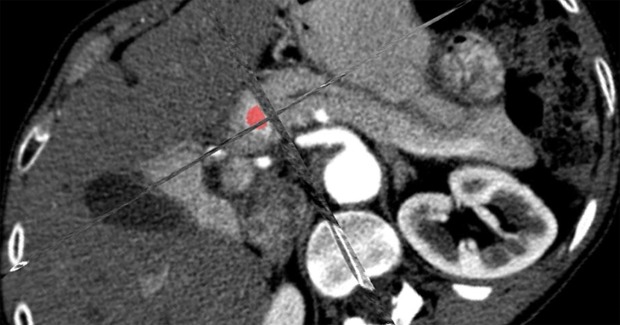
3D ROI within a small neuroendocrine isthmus pancreatic tumor on CT image on pancreatic phase for texture analysis.

In order to standardize CT examinations, all images were digitally reconstructed using a commercially available software (OsiriX Software, Pixmeo, Switzerland) with a slice thickness of 5 mm. Imaging data were then analyzed by a dedicated software for CT texture analysis (MaZda v4.6, Technical University of Lodz, Institute of Electronics, Poland). ROIs were drawn on the CT pancreatic phase image or on the CT image in which the tumor showed the greatest detectability and then copied to the corresponding images on CT pancreatic phase. Three dimensional (3D) ROIs were obtained by a manual segmentation of the tumor boundaries (Fig. 1). The following parameters were obtained: (a) Mean Value; (b) Variance; (c) Skewness; (d) Kurtosis; (e) Entropy.

### Statistical analysis

Tumors sizes were compared between tumor grades using Student’s T test. Categorical variables derived from the qualitative analysis were compared between tumor groups by using the χ2 test. Quantitative enhancement features and computed texture results were compared between groups using the Wilcoxon Mann-Whitman correlation test. Statistical analysis was conducted with commercially-available software (Analyse-it Software, v4.5.1 and Med Calc, Microsoft partner, v17.2). P values < 0.05 were considered statistically significant. Receiver operating characteristic (ROC) curves were calculated for significant parameters.

## Results

### Patients population and tumors

From our Institute archives, 304 patients affected by PanNEN were considered. From these, 204 patients were excluded due to un-availability of CT examinations, caused by damaged DICOM files or by old DICOM files not stored in the PACS. The 100 patients included were 55 males and 45 females, had a mean age of 54.8 years (range: 18–86 years). Histopathological diagnosis was obtained in 37 patients (37%) after surgical resection and with core-needle biopsy in 63 patients (63%). Tumor grade was G1 in 31, G2 in 52 and G3 in 17 cases. The tumor was in the pancreatic head in 42 patients, in the pancreatic body-tail in 52, and 6 patients had a diffuse involvement of the pancreatic gland. In 4 patients with multiple tumors, the biggest lesion was chosen for evaluation.

The mean tumor size was 44 mm (range 10–132 mm) for G1 tumors; 51.81 mm (range 8–130 mm) for G2; 52.35 mm (range 12–116 mm) for G3. No statistical significant differences were found for mean tumor size between groups (p > 0.05).

### Qualitative imaging analysis

**Figure 2:**
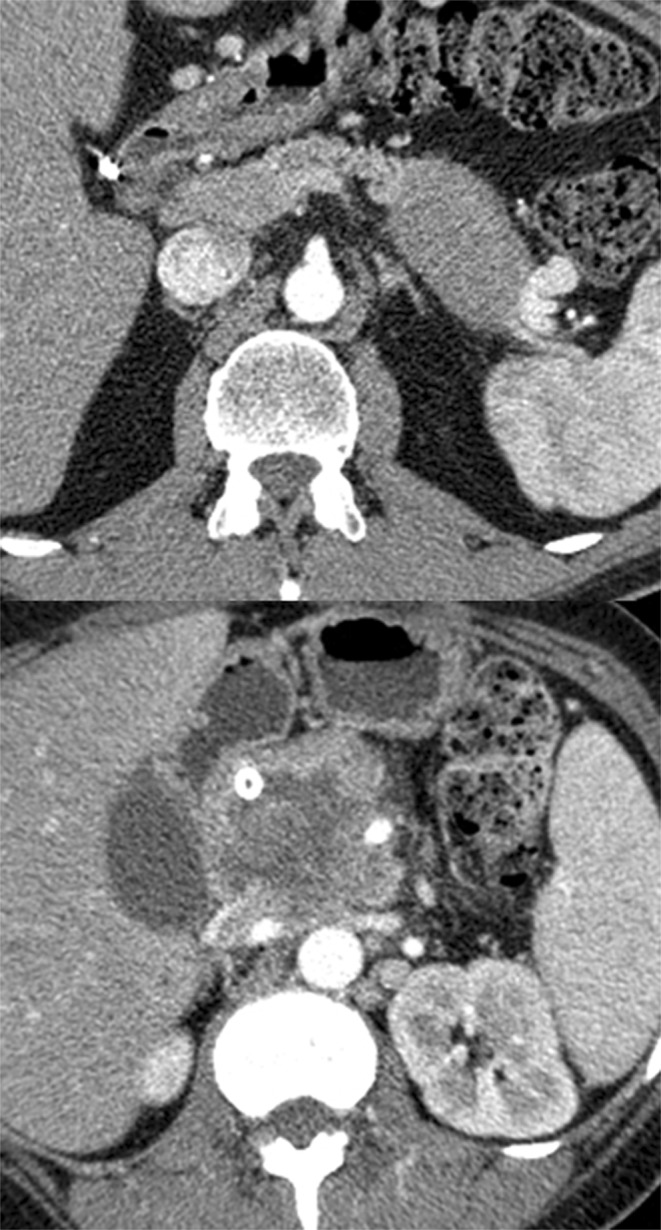
(**a**,**b**) neuroendocrine pancreatic tumor with sharp (**a**) or irregular (**b**) margins at CT.

Qualitative analysis results are reported in Table 1. Tumor margins were sharp (Fig. 2a) in 74 patients (74%); this feature was more common among G1 tumors (97%) compared with G2 and G3 tumors (69% and 47%, respectively). Tumor margins were irregular (Fig. 2b) in 26 patients (26%); this feature was significantly more common among G3 tumors (53%) compared with G1 and G2 tumors (3% and 31%, respectively). The frequency of irregular margins was significantly different between groups (p = 0.003). No significant differences between groups were found regarding the presence of hypodense areas, calcifications, upstream dilation of the MPD, CBD dilation, vascular involvement, liver metastases and enhancement.

**Table 1:** Qualitative imaging analysis results, reported separately for G1, G2 and G3 respectively.

	Sharp margin n Pts /100 (%)	Irregular margins n Pts /100 (%)	Hypodense areas n Pts /100 (%)	Calcifications n Pts /100 (%)	Dilation MPD n Pts /100 (%)	CBD n Pts /100 (%)	Mets n Pts /100 (%)	Vessels involvement n Pts /100 (%)	Pancreatic phase n Pts /100 (%)			Portal phase n Pts /100 (%)			Homo Enh n Pts /100 (%)	Hetero Enh n Pts /100 (%)
					Mean caliber mm				Hypo	Iso	Hyper	Hypo	Iso	Hyper		
G1	30	1	7	5	6	6	12	8	6	2	23	6	10	15	19	12
					6.2											
G2	36	16	16	18	11	5	32	35	23	2	27	16	9	27	19	33
					9.1											
G3	8	9	7	1	10	4	12	12	10	3	4	8	3	6	4	13
					13.1											
Tot	74	26	30	24	25	15	56	55	39	7	54	30	22	48	42	58

n: number; Pts: Patients; MPD: main pancreatic duct; CBD: common bile duct; Hypo: hypodense; Iso: isodense; Hyper: hyperdense; Tot: total of patients; Mets: metastases; Homo Enh: homogeneous enhancement; Hetero Enh: heterogeneous enhancement.

### Quantitative imaging analysis

Quantitative analysis results are reported in Table 2, where mean values, median and range of different enhancement ratio values and permeability ratios values are reported separately for G1, G2 and G3 groups respectively.

**Table 2:** Mean values and range of different enhancement ratio values and permeability indices in tumors divided according the tumor grade.

		Tumor parenchyma ratio 1	Tumor arterial ratio	Tumor parenchyma ratio 2	Tumor venous ratio	Tumor permeability ratio 1	Tumor permeability ratio 2	Tumor permeability ratio 3
G1	**Minimum**	0.540540541	0.170542636	0.702479339	0.4375	0.390180879	−0.417112299	0.634615385
	**Maximum**	3.23529411	0.909090909	3.323529412	0.975206612	1.401069519	0.114803625	1.847826087
	**Mean**	1.280183312	0.4434636	1.231802514	0.69781458	0.820234284	−0.074654369	1.187411125
	**Median**	1.171428571	0.4354	1.129374049	0.6966	0.8215	−0.05484	1.1441
G2	**Minimum**	0.520408163	0.169336384	0.429906542	0.298701299	0.389016018	−0.378600823	0.6375
	**Maximum**	4	0.905349794	5.733333333	0.907801418	1.453947368	0.138157895	1.775280899
	**Mean**	1.139382105	0.401851059	1.113379872	0.652510095	0.780080515	−0.033039593	1.082704628
	**Median**	1.009747126	0.3733	1.058823529	0.6563	0.7655	0.002193	0.9944
G3	**Minimum**	0.532786885	0.168831169	0.642857143	0.4	0.409375	−0.12	0.677083333
	**Maximum**	1.111111111	0.427083333	1.327731092	0.786069652	0.880434783	0.148867314	1.428571429
	**Mean**	0.765634679	0.284325474	0.906113192	0.539758797	0.622256058	0.033944598	0.923392464
	**Median**	0.736177885	0.2727	0.844320956	0.5132	0.6272	0.02273	0.8986

The mean HU_tumor_ on pancreatic phase images was 107.46, whereas the mean HU_tumor_ on portal phase images was 98.93. The mean HU_tumor_ of G1 tumors was 121.90 HU [range 60–200 HU] on pancreatic phase images and 103.83 [range 63–129] on portal phase images; the mean HU_tumor_ of G2 panNENs was 107.86 [range 51–220] on the pancreatic phase and 98.84 [range, 46–143] on the portal phase; the mean HU_tumor_ of G3 tumors was 79.94^62–129^ on the pancreatic phase and 89.4^69–158^ on the portal phase.

For all ratios, comparisons resulted significantly different between G1 and G3 groups (*tumor parenchyma ratio 1* p < 0.0001; *tumor parenchyma ratio 2* p = 0.0031; *tumor arterial ratio* p = 0.0007; *tumor venous ratio* p = 0.0003; *tumor permeability ratio 1* p = 0.0123; *tumor permeability ratio 2* p = 0.0026; *tumor permeability ratio 3* p = 0.0052), and between G2 and G3 groups (*tumor parenchyma ratio 1* p = 0.0003; *tumor parenchyma ratio 2* p = 0.0388; *tumor arterial ratio* p = 0.0015; *tumor venous ratio* p = 0.0017; *tumor permeability ratio 1* p = 0.0148; *tumor permeability ratio 2* p = 0.0338; *tumor permeability ratio 3* p = 0.0288), but not between G1 and G2 groups (*tumor parenchyma ratio 1* p = 0.0762; *tumor parenchyma ratio 2* p = 0.0247; *tumor arterial ratio* p = 0.1822; *tumor venous ratio* p = 0.1458; *tumor permeability ratio 1* p = 0.3142; *tumor permeability ratio 2* p = 0.1003; *tumor permeability ratio 3* p = 0.1337).

### Texture imaging analysis

Results of 3D CT texture analysis are reported in Tables 3 and 4. The mean texture values of G1 tumors were: Mean Value 202.01; Variance 5.07; Skewness −0.15; Kurtosis 0.07; Entropy 0.02. The mean texture values of G2 tumors were: Mean Value 343.94; Variance 4.69; Skewness −0.11; Kurtosis 0.44; Entropy 0.05. The mean texture values of G3 tumors were: Mean Value 186.63; Variance 5.5; Skewness 0.012; Kurtosis 1.88; Entropy 0.06.

**Table 3:** Mean values and range of 3D CT texture analysis parameters in tumors divided according the tumor grade.

		Mean Value	Variance	Skewness	Kurtosis	Entropy
G1	Minimum	65.344	1.103	−0.007679	−1.2813	0
	Maximum	2117.600	33.718	0.35643	0.65087	0.3981
	Mean	202.001581	5.07427097	−0.1521553	0.07386108	0.02500496
G2	Minimum	66.771	0.97089	−1.2643	−1.1067	0
	Maximum	2119.600	15.147	0.77918	2.541	0.50326
	Mean	343.942635	4.68824596	−0.1072736	0.4337585	0.04474871
G3	Minimum	67.087	1.0715	−0.81239	0.32116	0
	Maximum	2054.7	22.598	1.1562	17.665	0.21121
	Mean	186.629824	5.49595882	0.01199882	2.81655765	0.06323646

**Table 4:** Comparison among three-dimensional computed texture analysis parameters obtained in the three grading groups using the Mann-Whitney correlation test.

	G1 vs G2	G2 vs G3	G1 vs G3
Mean Value	p = 0.2123	p = 0.0842	p = 0.0113
Variance	p = 0.8580	p = 0.1032	p = 0.1922
Skewness	p = 0.4457	p = 0.3580	p = 0.2958
Kurtosis	p = 0.0063	p < 0.0001	p < 0.0001
Entropy	p = 0.2623	p = 0.0084	p = 0.0013

Kurtosis was significantly different among the three G groups (p = 0.0063 in G1 vs G2; p = 0.0004 in G2 vs G3 and p < 0.0001 in G1 vs G3) and Entropy differed significantly between G1 and G3 (p = 0.0013) and between G2 and G3 (p = 0.0084) tumors.

**Figure 3:**
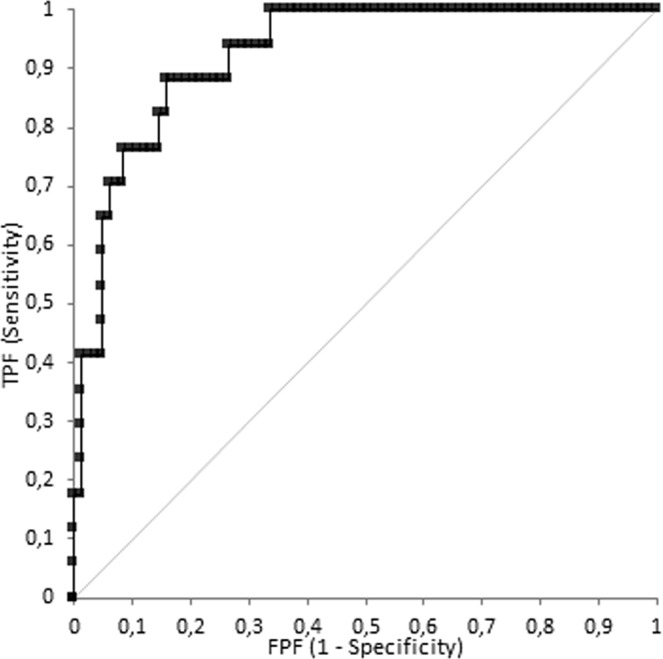
Receiver operating characteristic (ROC) curve for kurtosis giving AUC value of 0.924 for the diagnosis of G3 with a sensitivity and a specificity of 82% and 85% respectively by using 0.8 cut-off value.

**Figure 4:**
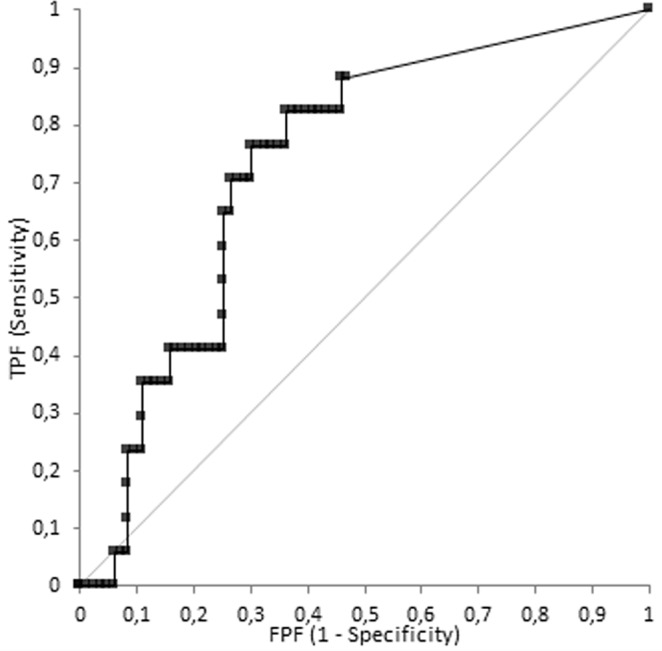
Receiver operating characteristic (ROC) curve for Entropy giving AUC value of 0.732 for the diagnosis of G3 with a sensitivity and a specificity of 82% and 64% respectively by using 0.002 cut-off value.

Receiver operating characteristic (ROC) curve was calculated for kurtosis giving AUC value of 0.924 (Fig. 3) for the diagnosis of G3 with a sensitivity and a specificity of 82% and 85% respectively by using 0.8 cut-off value. ROC curve was calculated for Entropy giving AUC value of 0.732 (Fig. 4) for the diagnosis of G3 with a sensitivity and a specificity of 82% and 64% respectively by using 0.002 cut-off value.

## Discussion

The aims of PanNENs imaging are detection, characterization and staging. To date, owing to technological developments, imaging could cover new significant roles, such as evaluation of tumor aggressiveness and prognostic prediction by assessing earlier therapeutic response^14^. Texture analysis that evaluates pixels values, variations and distribution using comparable parameters, has shown promising results in predicting tumor pathological features^13,15–21^, overall survival^13,21–23^, relapse risk^24,25^, and response to therapy^26,27^ for different tumors in different organs. To date, there is no study on the role of texture analysis for pancreatic neuroendocrine neoplasms in Literature. In this study a CT qualitative, quantitative and texture analysis of PanNENs was performed.

At qualitative analysis, only tumor margins resulted to be a useful parameter. Qualitative analysis also showed differences in the most frequent presentation according to tumor grades. G1 tumors had more frequently sharp margins, no hypodense areas and no calcifications within the lesion, no dilation of main pancreatic duct and common bile duct, no liver metastases, no involvement and/or infiltration of peri-pancreatic vessels, homogeneous enhancement, hyperdensity in both pancreatic and portal phases. G2 tumors most frequently presented with sharp margins, no hypodense areas and no calcifications within the lesion, no dilation of main pancreatic duct and common bile duct, they could have liver metastases, involvement and/or infiltration of peri-pancreatic vessels, heterogeneous enhancement, and they were hyperdense in both pancreatic and portal phases. G3 tumors had most frequently irregular margins, no hypodense areas and no calcifications within the lesion, dilation of main pancreatic duct but not of common bile duct, liver metastases, involvement and/or infiltration of peri-pancreatic vessels, heterogeneous enhancement, and hypodense in both pancreatic and portal phases.

The quantitative analysis with enhancement ratios and permeability indexes permitted to distinguish G1 from G3 tumors and G2 from G3 tumors, while it did not permit to differentiate G1 from G2 tumors. These results are in general agreement with those present in Literature. Several studies showed correlations between imaging features of PanNENs, especially the type of enhancement, and histological findings^5–11^. With respect to other tumors, such as lung or breast cancers where a rich angiogenesis can be a predictive value of poor outcome^28^, the correlation between vascularization amount and aggressiveness in PanNENs is different. Overtly malignant and high-grade tumors have an altered vascularization, resulting in an atypical contrast enhanced pattern, such as hypovascularity in arterial phase or late enhancement in venous phase^29^. Belousova *et al*.^5^ concluded in their study that tumor size >2 cm, arterial enhancement ratio <1.1 (tumor to pancreas CE value in arterial phase), and late contrast enhancement were indicative of G2 tumors, and this information could be used to support decisions considering the extent of tumor resection or the possibility of a conservative approach allowing for individualized decision making. Cappelli *et al*.^6^ reported that contrast enhancement pattern of PanNENs, as determined during multiphasic study, correlated with histological grading, allowing to predict their biological behavior. In their study, a lesion showing type A pattern of contrast enhancement (early CE in arterial phase and rapid wash-out resulting hypodense in portal phase) can be reasonably considered as benign; on the contrary type B2 (delayed CE in venous and late phases) should be considered strongly suggestive of malignancy. Kim *et al*.^7^ reported that G3 neuroendocrine carcinomas had characteristic CT features, such as portal enhancement ratio <1.1, poorly defined margin, tumor size >3 cm, bile duct dilation, and vascular invasion. Consequently, they concluded that when these CT findings are used in combination, G3 NECs can be differentiated from G1and G2 NETs with high diagnostic accuracy. Luo *et al*.^9^ showed that multi-slice computed tomography imaging is a feasible technique for predicting the pathological classification of PanNENs: peri-pancreatic tissue or vascular invasion and lesser enhancement at the arterial phase were significantly correlated with higher grade PanNENs. Takumi *et al*.^10^ found that a combination of CT features, including tumor size (≥20 mm), metastases and tumor conspicuity led to an increased diagnostic accuracy for G2 PanNENs, compared to each approach alone. They found that a larger tumor size (≥20 mm) and non-hyperattenuation during the portal/venous phase were associated with G2 PanNENs, suggesting that contrast-enhanced CT features may help predict the pathological tumor grades. Yamada *et al*.^11^ affirmed in their study that the degree of CT enhancement in the pancreatic phase was a significant predictor of G2 PanNENs.

In the present study, CT texture analysis discerned G1 from G2 and G3 tumors, and G2 form G3 tumors with a statistically significant difference. The most important CT texture analysis parameter resulted to be Kurtosis, but also Entropy values resulted significantly different in the distinction of G1 from G3 tumors and G2 from G3 tumors. As a consequence, CT texture analysis has a superior performance in the prediction of PanNEN tumor grade, in comparison to qualitative and quantitative analysis.

This analysis proved to be useful in studying different tumors in other organs. Liu S *et al*.^15^ reported that CT texture analysis held great potential in predicting differentiation degree, Lauren classification and vascular invasion status of gastric cancers. Yasaka *et al*.^16^ showed that using CT quantitative texture analysis high-risk thymic epithelial tumor can be differentiated from low-risk ones with a high diagnostic performance. Liu Y *et al*.^17^ revealed in their study that texture analysis on contrast enhanced CT images could be helpful in predicting pathologic grade of lung adenocarcinoma. Yu *et al*.^18^ concluded their study affirming that texture analysis is a promising non-invasive tool for distinguishing renal tumors on CT images. Hodgdon *et al*.^19^ affirmed that CT texture analysis can be used to accurately differentiate fat-poor angiomyolipoma from renal cell carcinoma on unenhanced CT images. Zhang *et al*.^20^ proved that CT texture analysis is a feasible tool for differentiating low-grade urothelial carcinoma from high-grade ones.

Our data suggest that CT texture analysis could have an oncologic application in the clinical practice reporting data not visible but present in the diagnostic CT images, as already stated in Literature^14^. Choi *et al*.^30^ found interesting results in distinguishing PanNENs G1 from G2/G3 by using CT texture analysis. In the paper the majority of the included tumors were G1 (n = 45) whereas there were few G3 tumors (n = 5). Moreover, for Kurtosis calculation the CT portal phase were analyzed. Some differences in the results in respect to our study are therefore expected. Of the 100 PanNENs included in our study, 31 were G1, 52 were G2 and 17 were G3, and moreover no statistical significant difference in tumor dimensions were found in the study population. 3D texture analysis was performed in all cases by using CT arterial phase that represents the best dynamic phase able to potentially highlight the tumor arterial network. Therefore, our results seem to come from a correct evaluation of a well distributed study population. In the present study statistical significant difference were found in the Entropy between G1 and G3 and G2 and G3 and in the Kurtosis between G1 and G2, G2 and G3, G1 and G3. In particular, in the present study G3 tumors showed higher values of Kurtosis and this is graphically represented by a leptokurtic distribution related to the more presence of intra-tumoral fibrosis/necrosis. On the opposite G1 tumors Kurtosis results are graphically represented by a platykurtic distribution related to the more homogeneous representation of all tissue components, in many cases as in the pancreatic parenchyma.

A possible clinical scenario can be supposed: in the same moment that a neuroendocrine neoplasm is detected, characterized and staged, texture analysis could provide tumor grade prediction, allowing better patient management.

Limitations of our study were the retrospective design of the study and the inclusion of core-needle biopsies together with resected specimens for the final pathological diagnosis and tumor grade evaluation. Prospective studies are expected in order to confirm present results, clarifying possible differences with previous or other studies in this field.

## Conclusion

Quantitative CT evaluation of PanNENs can predict tumor grade, discerning G1 from G3 and G2 from G3 tumors. CT texture analysis can predict PanNENs tumor grade, distinguishing G1 from G3, G2 from G3, and G1 from G2 tumors. CT texture analysis parameters therefore could be a useful surrogate for neuroendocrine pancreatic neoplasms grading.
